# Supplementary material for: A Global Screen for Assembly State Changes of the Mitotic Proteome by SEC-SWATH-MS
Source: Cell Syst. 2020 Feb 26;10(2):133–155.e6. doi: 10.1016/j.cels.2020.01.001 (PMC7042714; doi:10.1016/j.cels.2020.01.001)

Q9NRR4 | RNC_HUMAN | DROSHA RN3 RNASE3L RNASEN
Monomer MW [kDa]: 159.316 Monomer expected elution fraction: 36

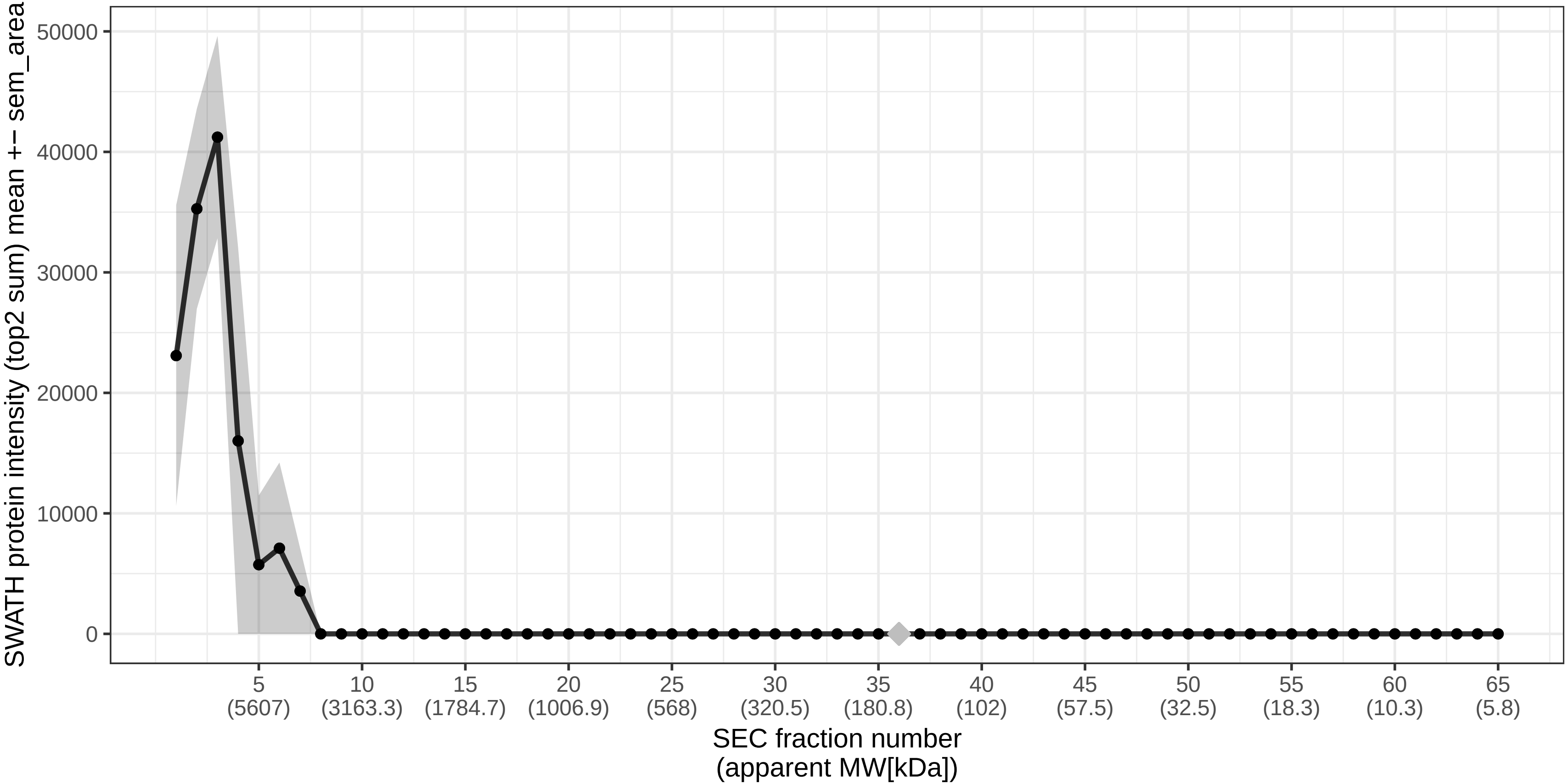

Supplement: Data S1. SEC-SWATH-MS Protein Chromatograms, Related to Figure 1 [file mmc6.zip › SECchrom_Q9NRR4_RNC_HUMAN_DROSHA_RN3_RNASE3L_RNASEN.pdf]
